# Custodiol HTK versus Plegisol: in-vitro comparison with the use of immature (H9C2) and mature (HCM) cardiomyocytes cultures

**DOI:** 10.1186/s12872-022-02536-6

**Published:** 2022-03-17

**Authors:** Rafał Nowicki, Mikołaj Berezowski, Julita Kulbacka, Katarzyna Bieżuńska-Kusiak, Marek Jasiński, Jolanta Saczko

**Affiliations:** 1 Clinical Department of Cardiac Surgery, Wroclaw Medical University, Wroclaw, Poland; 2 Doctoral School, Wroclaw Medical University, Wroclaw, Poland; 3 Department of Molecular and Cellular Biology, Faculty of Pharmacy, Wroclaw Medical University, Wroclaw, Poland; 4 Children’s Memorial Pediatric Health Institute, Warsaw, Poland

**Keywords:** Cardioplegia, Cardiomyocyte cultures, Oxidative stress

## Abstract

**Background:**

Although cardioplegia is used since the ‘70s of the last century, debate on cardioprotection during cardio-surgical procedures is still actual. The selection of a particular method depends mainly on the preferences and experience of a specific center or even surgeon. Crystalloid cardioplegia is an aqueous ion solution similar to intracellular (Custodiol HTK) or extracellular (Plegisol) fluid. The potensional clinical advantages of relatively new idea of cardioplegia solution based on intracellular composition (Custodiol HTK) justifies futher research, but only a few used cultured cells in laboratory conditions.

**Methods:**

In this study, the authors sought to compare Custodiol HTK with Plegisol cardioplegia solutions using an in-vitro model simulating cardioplegic arrest. The efficacy of myocardial protection during ischemia was investigated with susceptible indicators like the appearance of the deleterious effect of reactive oxygen species and oxidative stress markers. Immersed human cardiomyocytes and rat cardiomyoblasts H9C2 in cardioplegia for 4 h were examined for expression of oxidative stress markers (MnSOD, iNOS, HSP27), cardioplegic solutions cytotoxicity, and peroxidation damage of the cell’s lipids and proteins. All tests were performed after 0.5 h, 1 h, 2 h, and 4 h of incubation in identical physical and biological conditions, which is difficult to achieve in clinical trials.

**Results:**

The lower cytotoxicity index performed on matured cells of human cardiomyocytes and highest dehydrogenase level showed after incubation with Custodiol HTK. This did not apply to tests on immature cells H9C2. Custodiol HTK induced significantly stronger iNOS expression. The decrease of HSP27 concentration has been instantaneous and maintained troughout the study only in both cultures incubated with Custodiol HTK. The other tests: lipid peroxidation, carbonyl groups concentration and MnSOD expression show no clear superiority evidence of used cardioplegic solutions.

**Conclusions:**

Considering proceeded examinations on cultured cardiomyocytes, Custodiol HTK appears to be safer than Plegisol.

## Background

Cardiac surgery on the arrested heart with cardiopulmonary by-pass (CPB) has been a routine and safe method of treatment of numerous congenital and acquired heart diseases for dozens of years. However, the cessation of coronary circulation involves the risk of ischemia and post-ischemic (reperfusion) injury in the form of oxidative stress (OS) [1]. OS depends on the toxic effect of reactive oxygen species (ROS) during reperfusion time and their affinity to the cell structures, including lipids, proteins, and nucleic acids [2]. The oxidative reaction leads to cell malfunctions or even structural cell damage where the consequence is reversible or irreversible heart dysfunction (insufficiency).

In order to minimize the consequences of myocardial ischemia/reperfusion injury during cardio-surgical procedures, a number of protection methods have been introduced. The most important one is the administration of cardioplegia. The currently used cardioplegic solutions are divided into two groups, the first one, blood cardioplegia, is based on the patient’s blood and the other one, the crystalloid cardioplegia, is an aqueous ion solution. Its formula is similar to the intracellular or extracellular fluid. Blood cardioplegia is obtained by mixing the patient’s blood with crystalloid cardioplegia by a ratio of 4:1 or 8:1, another method is adding potassium ions directly to blood (e.g., Calafiore protocol) [3, 4]. Cardioplegia is administered to the aortic root, directly to coronary ostia (antegrade), or coronary sinus (retrograde) at normothermia in hypothermia. The protective effect of cardioplegia consists of quick cardiac arrest in diastole by cell membrane depolarization and suppressing its mechanical and electric activity. The currently applied cardioplegic solutions are based on depolarizing abilities of potassium ions; however, researches are conducted on low-potassium cardioplegia using sodium and magnesium ions or other non-depolarizing substances. At present, there is no universal method of heart muscle protection [1]. The selection of a particular method depends mainly on the preferences and experience of a particular center or surgeon.

Although numerous publications compare different types of cardioplegic solutions, only a few of them used cultured cells in laboratory conditions. Our in vitro study aimed to evaluate the efficacy of two well-known crystalloid cardioplegic solutions on cultured cardiomyocytes. The appearance of oxidative stress markers and damage of cultured cardiomyocytes after simulating the cardioplegic arrest were determined.

## Methods

### Cells cultures

Two cardiac cell lines were applied for in-vitro experiments. Rat cardiomyoblasts, immature cells—H9C2 (obtained from the Lab of Department of Medical Biochemistry, Wroclaw Medical University) which were grown in Dulbecco's Modified Eagle's Medium glucose (Lonza) with the addition of 10% fetal bovine serum (FBS, Lonza), 2 mM Glutamine and 100 × penicillin/streptomycin (Sigma). Primary human cardiac myocytes (HCM, PromoCell GmbH, Germany) were isolated from the ventricles of the adult heart and grown in Myocyte Growth Medium (C-22070, PromoCell GmbH, Germany) with the addition of Supplement Mix (C-39275, PromoCell GmbH, Germany). For experiments, the cells were removed by trypsinizing (0.25% Trypsin–EDTA solution, Sigma), and washed with PBS. The cells were maintained in a humidified atmosphere at 37 °C and 5% CO_2_.

### Cardioplegia

Custodiol HTK (Kӧhler Chemie GmbH, Germany) is intracellular ionic composed cardioplegia (low K^+^) with the addition of three amino acids (histidine, tryptophan, ketoglutarate) and based on Bretschneider’s solution introduced in 1975 [5]. Custodiol HTK based on its buffering capacity, is often used as a single-dose product.

Plegisol (Hospira Inc, USA) based on STH N°2 (St. Thomas’ Hospital, London) extracellular solution (high K^+^) as a multi-dose solution is used from the ‘70 s of the last century [6]. Plegisol requires buffering with the NaHCO_3_ just before administration. The compositions of both solutions are shown in Table 1.

**Table 1:** The composition of Custodiol HTK and Plegisol (after buffering with NaHCO_3_)

Composition [mmol/L]	Cardioplegia	
	Custodiol HTK	Plegisol (STH-2)
K^+^	9.0	16.0
Na^+^	15.0	120.0
Mg^++^	4.0	16.0
Ca^+^	–	1.2
NaHCO_3_ [mEq/L]	–	10.0
pH	7.1–7.2	7.8
Osmolality [mosM/KgH_2_O]	295–325	280
Adds	Histidine Tryptophan Ketoglutarane Mannitol	–

### Simulation of the cardioplegic arrest and OS

Cells were removed from medium and immersed in cooled (4–6 °C) both cardioplegic solutions incubated for 0.5, 1, 2, and 4 h in a humidified atmosphere at 37 °C and 5% CO_2_. After that, the experiments were performed. The temperature of cardioplegic solutions and the specific period of incubation were similar to the intraoperative conditions. Thus, we simulated the ischemia, such as during the aorta cross-clamping (arrest of the coronary perfusion). However, the typical reperfusion injury was not possible to obtain in our in-vitro model we provoked OS. The imbalance of prooxidant and antioxidant levels resulting from ischemia resulted in the overproduction of reactive oxygen species (ROS). However, the estimation of ROS concentration cannot be measured, the deleterious effect of ROS affected cellular lipids, proteins, and cardiomyocytes viability was examined in viability assay, the concentration of malondialdehyde (MDA) as an evidence of lipid peroxidation and determination of carbonyl groups caused by proteins damage. The presence of OS was also proven by the changes in antioxidative enzyme activity (iNOS, MnSOD) and chaperone activity protein (HSP27) which are regarded as OS markers.

### The viability assay and cytotoxicity

The viable cardiomyocytes contain NAD(P)H-dependent oxidoreductase (mitochondrial dehydrogenase) enzyme, which reduces MTT (tetrazolium salt (3-(4,5-dimethylthiazol-2-yl)-2,5-diphenyltetrazolium bromide) to insoluble formazan crystals. According to the protocol procedure, the cellular viability and the cytotoxic impact of cardioplegic solutions were determined by the MTT assay (Sigma, In VITRO Toxicology Assay). Cells were seeded into 96-well microculture plates (Nunc, Biokom) and allowed to attach for 24 h. Then the incubation with Custodiol HTK or Plegisol for 0.5 h, 1 h, 2 h, and 4 h was performed. The dissolved and colored formazan crystals were quantified spectrophotometrically by measuring the absorbance at 570 nm (Multiscan MS microplate reader, Theremo Fisher). The results were expressed as a percentage of the untreated control (percentage of control cells).

### Protocol for cells preparation for lipid peroxidation and carbonyl groups

Cells were grown in 25 cm^2^ flasks (Nunc) to obtain full monolayer. Then they were incubated with Custodiol HTK or Plegisol for 0.5 h, 1 h, 2 h, and 4 h. After the treatment, cells with cardioplegia in-vitro were removed by trypsinizing and washed twice in PBS (IITD, PAN, Poland). Then cells suspensions (ca. 5 × 10^6^) were centrifuged 5 min at 1500 rpm (Centrifuge MPW Med. Instruments MPW-341 with stable rotor).

### Lipid peroxidation

Cell samples were suspended in 200 µL of PBS. Then 200 µL of 15% TCA (trichloric acid; Roth) in 0.25 M HCl and 200 µl of 0.37% TBA in 0.25 M HCl were added. The control sample contained 200 µL of deionized water instead of the cell suspension. Then the samples were incubated for 20 min at 90 °C (Termoblock TB-941 U). After incubation, the samples were centrifuged for 5 min at 5000 rpm. MDA, the final product of fatty acid peroxidation, reacts with TBA to form a colored complex. The level of TBARS was measured based on the absorbance at the wavelength of 535 nm. The concentration of malondialdehyde was quantified spectrophotometrically based on a set of MDA standards of known concentration. All measurements were performed on UV/Vis spectrophotometer (JASCO V-530, MEDSON).

### Proteins’ damage—carbonyl groups

Oxidative damage to proteins was investigated by determining carbonyl groups based on the reaction with dinitrophenylhydrazine (DNPH) (Sigma). Briefly, proteins were precipitated by the addition of 20% trichloroacetic acid (Sigma) and dissolved in 10 mM DNPH, and the absorbance was read at 570 nm. The results were calculated using an extinction coefficient of ε = 21.01 mmol^−1^/cm^−1^ for aliphatic hydrazone. Experiments were repeated three times.

### Immunocytochemistry (MnSOD, iNOS, HSP27)

Manganese superoxide dismutase (MnSOD) is a typical antioxidative enzyme located in mitochondria. It plays a crucial role in ROS scavenging and protecting mitochondria from oxidative damage. Inducible nitric oxide synthase (iNOS) catalyzes nitric oxide production, which prevents ROS formation and activates the synthesis of glutathione—another antioxidative factor. Heat shock protein 27 (HSP27) is regarded as a protein chaperone, antioxidant, and inhibits apoptosis. Immunocytochemistry was performed using the ABC method. Briefly, cultures were fixed and dehydrated using 4% paraformaldehyde and an ethanol gradient respectively. Samples were then permeabilized and blocked by incubation with 0.1% Triton X-100 (Sigma) in PBS. The expression of proteins was visualized with the mouse monoclonal antibody (1:100, Santa Cruz). For conventional bright-field microscopy (peroxidase-ABC labeling), the samples were incubated with the diaminobenzidine-H_2_O_2_ mixture (DAKO) to visualize the peroxidase label, counterstained with hematoxylin (Alchem, Poland) for 30 s. Samples were examined on a simple Olympus microscope (BX41, Japan). Stained cell numbers were determined by counting 100 cells in 3 randomly selected fields. Two independent investigators performed the counting. The result was judged to be positive if staining was observed in more than 5% of cells. The intensity of immunocytochemical staining was evaluated as (−) negative (no reaction), (+) weak, (++) moderate, and (+++) strong. All experiments were repeated three times.

### Statistical analysis

The normality of the continuous variables was checked by the Shapiro–Wilk test. The significance of the difference between mean values of different groups of cells (MTT test, lipid peroxidation, carbonyl groups concentration, MnSOD, iNOS and HSP27 expression) compared with the control group untreated cells) was assessed by Student’s t-test with value of *p* ≤ 0.05 taken as statistically significant. Statistical analysis was performed using STATISTICA 10 software.

## Results

### Cell viability

**Fig. 1:**
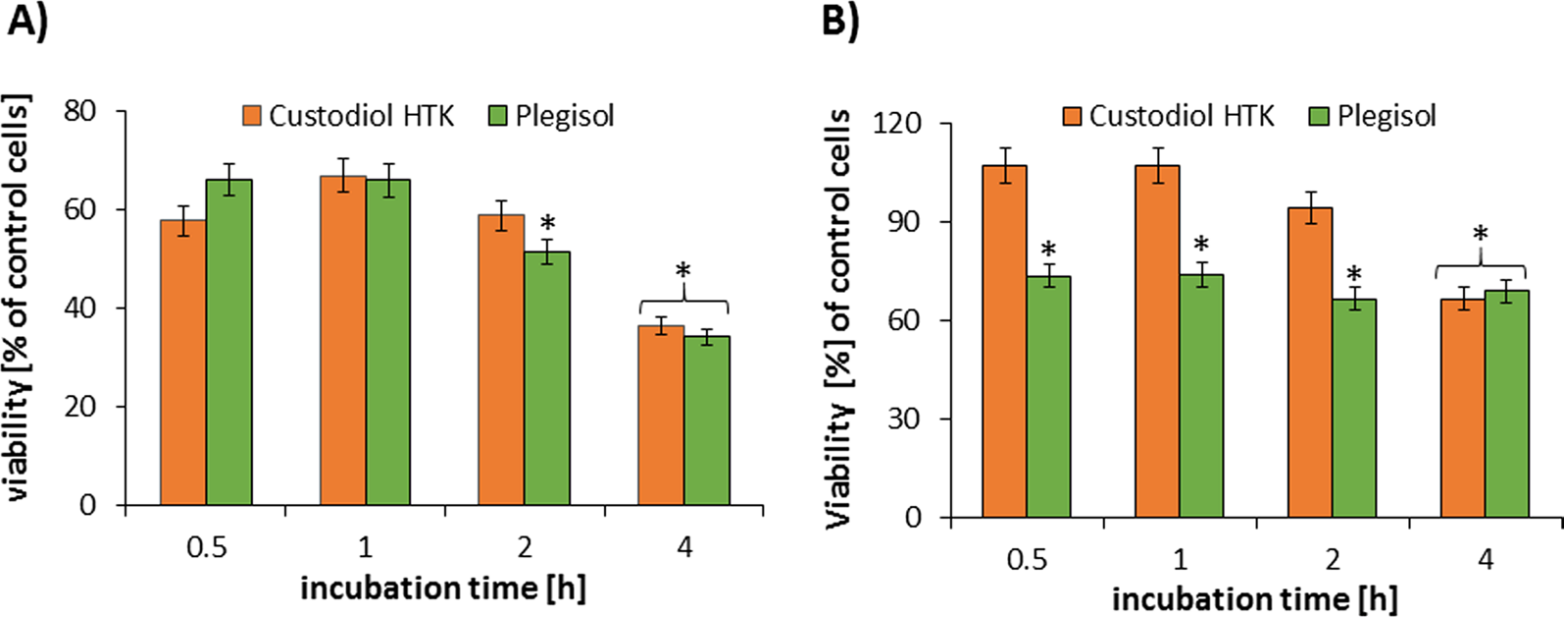
The viability assay after incubation with cardioplegia in **A** rat H9C2 cells and **B** human HCM cells. The results are averaged. The standard deviation is marked with the error bar. *statistically significant for *p* < 0.05

Both solutions did not cause a decrease of cytotoxicity index under 50% up to the second hour of incubation time (Fig. 1). A significant decrease was observed after four hours of incubation for both cardioplegic solutions. However, during the first 2 h of incubation with Custodiol, viability of HCM was above 90% compering the control cells and significantly lower in the Plegisol group (60–70%) (Fig. 1B). The cytotoxicity of cardioplegias grew with the time of incubation.

### Lipid peroxidation

**Fig. 2:**
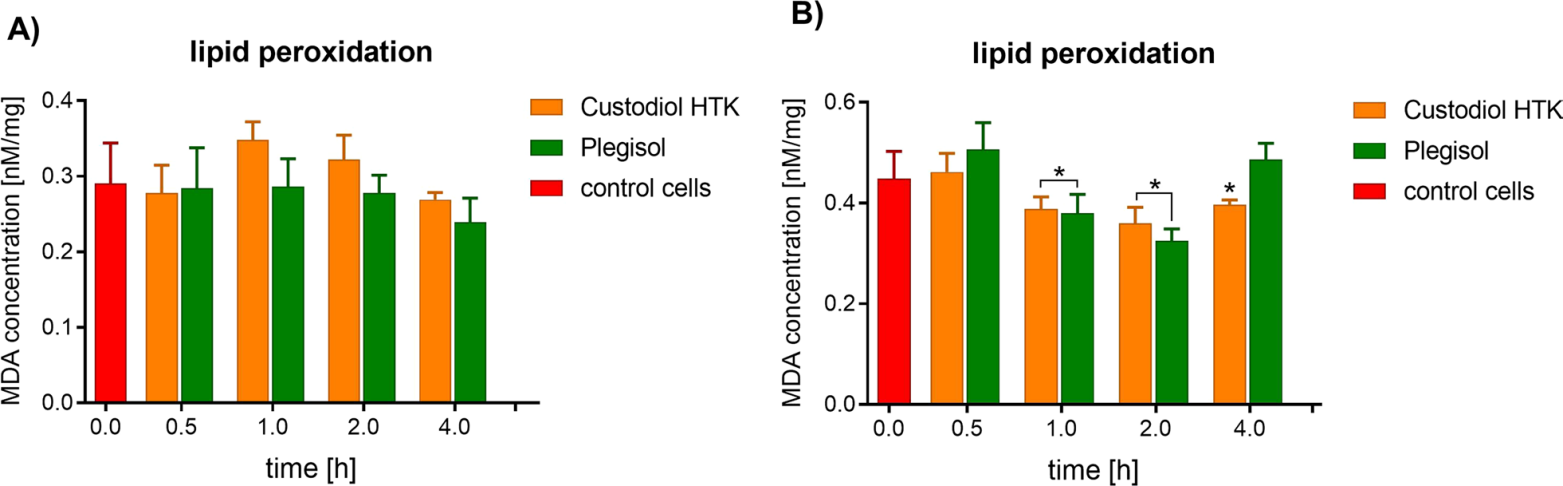
The evaluation of lipid peroxidation by malondialdehyde (MDA) concentration level in **A** rat H9C2 cells and **B** human HCM cells after incubation with cardioplegia. The results are averaged. The standard deviation is marked with the error bar. *statistically significant for *p* < 0.05

In comparison to the control cells, only Custodiol HTK induced a slight increase of lipid peroxidation from 1 to 2 h of incubation (Fig. 2). After 4 h the MDA concentration dropped under control level. Plegisol did not provoke lipid peroxidation in H9C2 cells during every applied incubation time. Custodiol HTK did not provoke lipids peroxidation in HCM cells. The MDA concentration was below the control level in samples after 1 h, 2 h, and 4 h of incubation with both cardioplegic fluids. Only after 0.5 h of incubation with Plegisol, a slightly increased level of lipid peroxidation was observed (*p* = 0.000041), however MDA level was comparable to the control level.

### Proteins’ damage (carbonyl groups concentration)

**Fig. 3:**
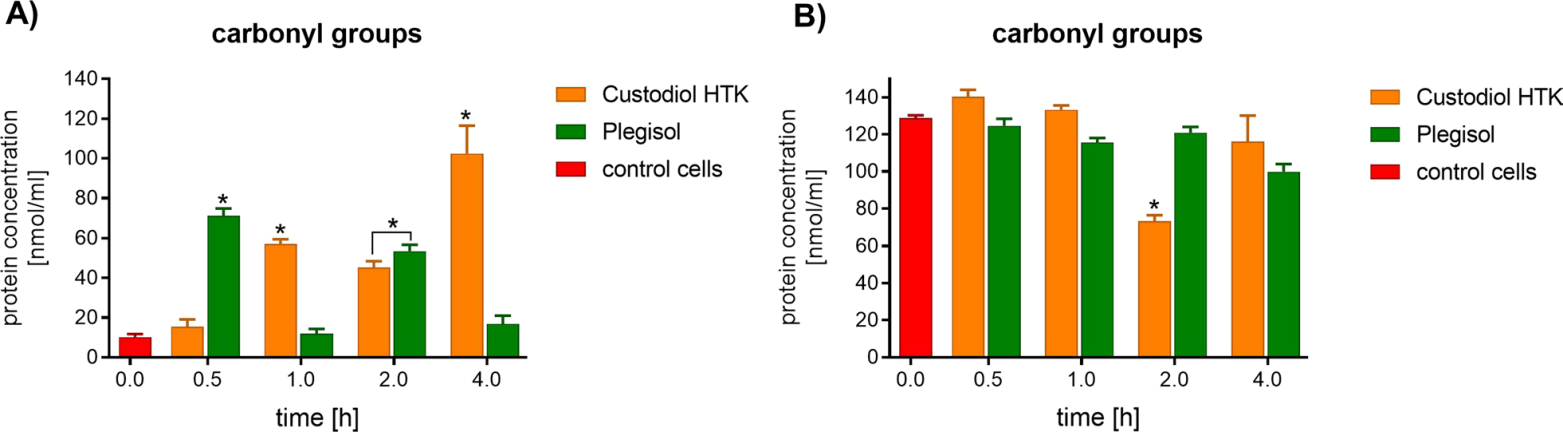
The evaluation of protein damage by carbonyl group concentration level in **A** rat H9C2 cells and **B** human HCM cells after incubation with cardioplegia. The results are averaged. The standard deviation is marked with the error bar. *statistically significant for *p* < 0.05

Both cardioplegic solutions generated a statistically significant increase in carbonyl groups in H9C2 cells (Fig. 3). A significant increase of carbonyl group level was observed after 0.5 h and 2 h of incubation with Plegisol solution and after 1 h and 4 h with Custodiol HTK. Moreover, the raised level of carbonyl group concentration was independent on the time of incubation. The highest protein damage in H9C2 cells was after 4 h of incubation with Custodiol HTK. The concentration of carbonyl groups in HCM cells showed a slight fluctuation comparing control cells. After 2 h of incubation with Custodiol HTK, the lowest concentration of carbonyl groups was observed.

### The evaluation of MnSOD

**Fig. 4:**
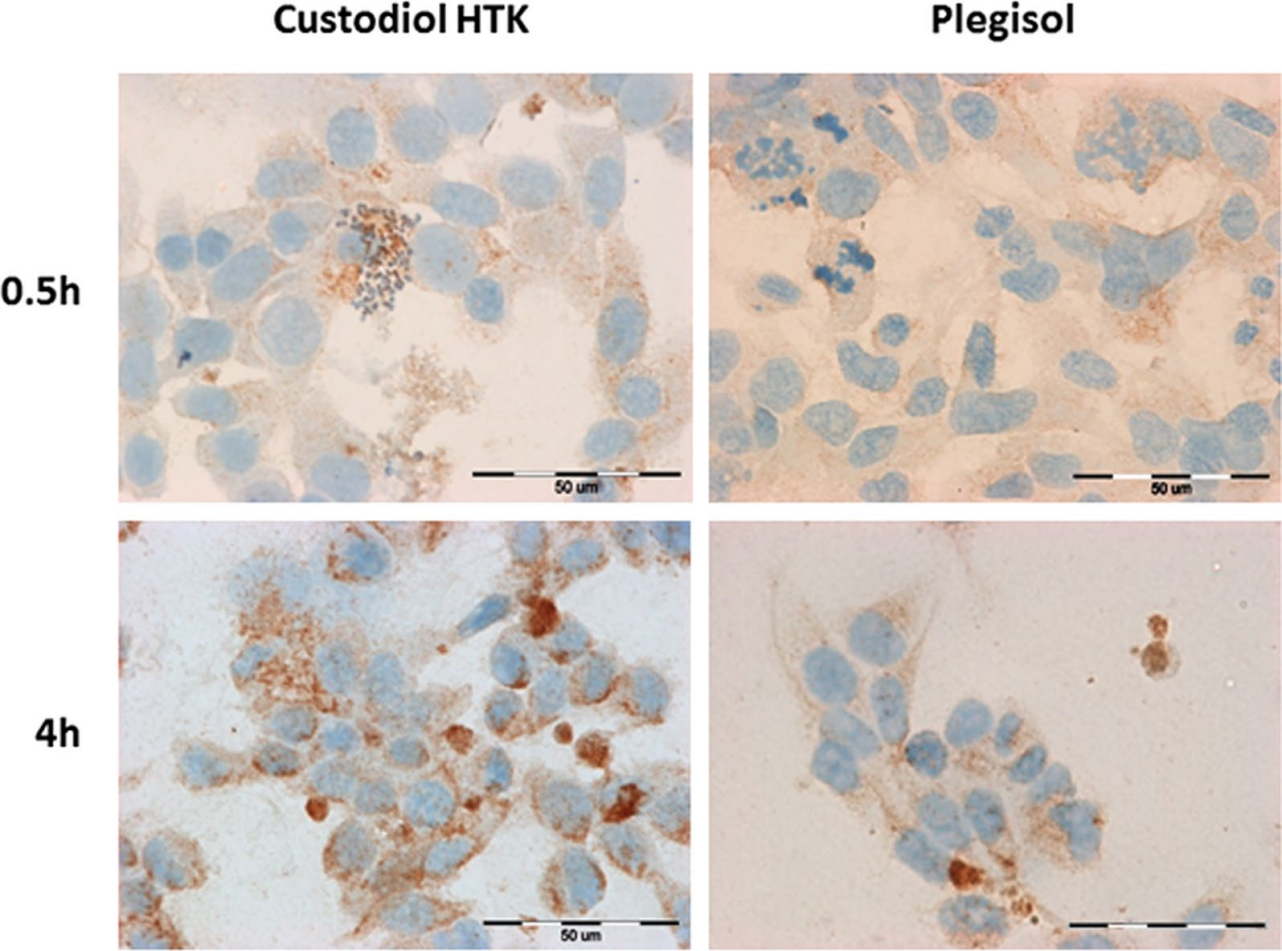
The evaluation of MnSOD immunocytochemical reaction in rat H9C2 cells after 0.5 h and 4 h of incubation with Custodiol HTK or Plegisol

**Fig. 5:**
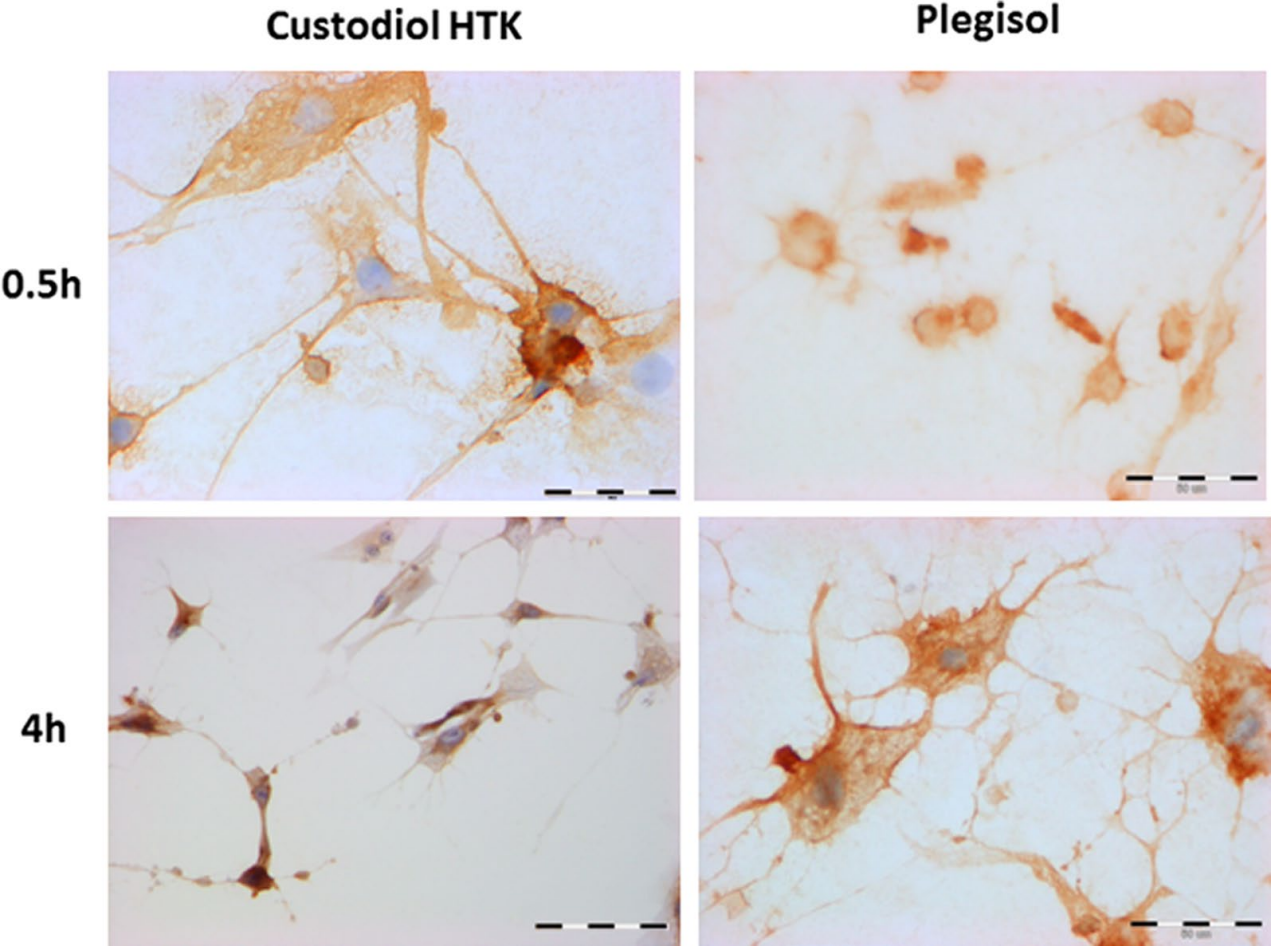
The evaluation of MnSOD immunocytochemical reaction in human HCM cells after 0.5 h and 4 h of incubation with Custodiol HTK or Plegisol

The immunocytochemical analysis of MnSOD in cardiac cells is presented in Table 2A, Fig. 4 (H9C2) and Table 2B, Fig. 5 and (HCM). The intensity of the reaction and the percentage of stained cells were similar in both examined cardioplegic solutions at the beginning and the end of incubation. A non-significant increase was observed after 1 h of incubation with Custodiol HTK (95%) in comparison to incubation with Plegisol (70%). MnSOD expression in HCM cells was significantly higher than in control cells. Any correlation between incubation time, the number of stained cells, reaction intensity, and the type of cardioplegia was not observed. A slightly stronger reaction was noticed in cells suspended in Plegisol after 0.5 h and 1 h compared to cells incubated with Custodiol HTK.

**Table 2:** The immunocytochemical evaluation of MnSOD stained reaction in: (A) H9C2 cells (B) HCM cells

Incubation time [h]	Custodiol HTK	Plegisol	Control cells
**(A)**			
0.5	67%* +/++	69%* +	10% +
1	70%* +/++	95%* ++	
2	96%* ++	95%* ++	
4	95%* ++	98%* ++	
**(B)**			
0.5	80%* ++	99%* ++/+++	10% +
1	85%* ++/+++	98% +/++	
2	97%* ++/+++	77%* +	
4	98%* +/++	98%* ++/+++	

*for *p* ≤ 0.001

### The evaluation of iNOS

**Fig. 6:**
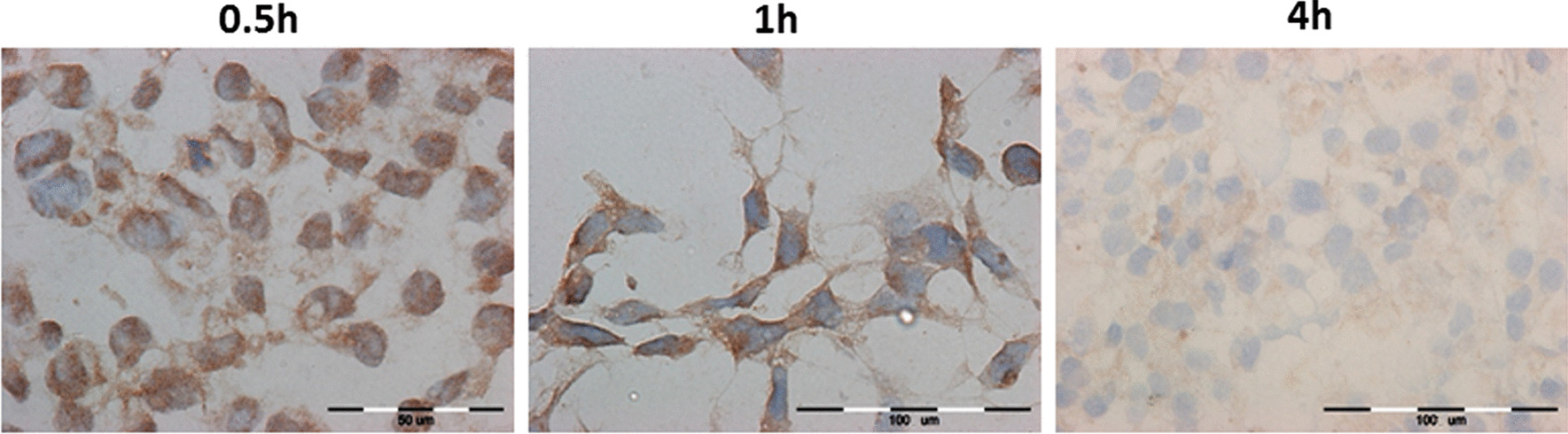
The evaluation of iNOS immunocytochemical reaction in rat H9C2 cells after 0.5 h, 1 h and 4 h of incubation with Plegisol

**Fig. 7:**
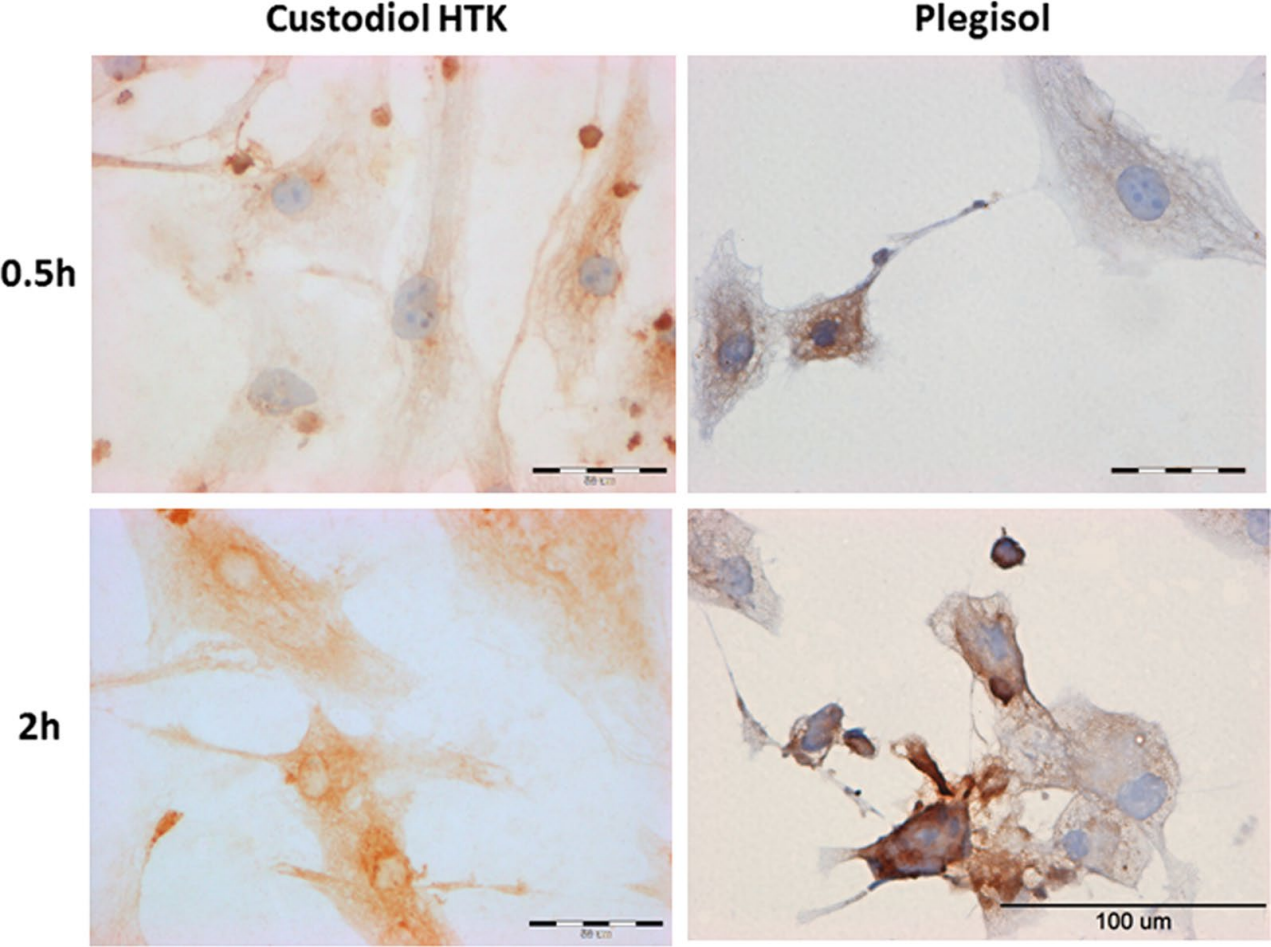
The evaluation of iNOS immunocytochemical reaction in human HCM cells after 0.5 h and 2 h of incubation with Custodiol HTK or Plegisol

The immunocytochemical evaluation of iNOS in cardiac cells is presented in Fig. 6 for rat H9C2 after 0.5 h, 1 h and 4 h of incubation with Plegisol and all the results for H9C2 in Table 3A. In HCM cells, the effects of immunocytochemical study are shown in Fig. 7 after 0.5 h and 2 h of incubation with Custodiol HTK or Plegisol and in Table 3B. The slight increase of iNOS expression was observed after 0.5 h, 1 h, and 2 h of incubation in H9C2 cells for both Custodiol and Plegisol solutions. At 4 h iNOS expression increased up to 20% (Custodiol HTK) and 25% (Plegisol) of H9C2 cells. Custodiol HTK activated significantly higher iNOS expression in HCM cells than Plegisol and control cells. Seventy percent of cardiomyocytes incubated with Custodiol HTK has been stained already after 0.5 h. The expression increased up to 95% after 2 h with strong reaction intensity (+++). iNOS expression suspended in Plegisol increased statistically considerably to 85% only after 2 h of incubation and dropped to 25% stained cells after 4 h.

**Table 3:** The immunocytochemical evaluation iNOS expression in: (A) H9C2 cells, (B) HCM cells

Incubation time [h]	Custodiol HTK	Plegisol	Control cells
**(A)**			
0.5	< 5% −/+	< 5% −/+	0
1	< 5% −/+	< 5% −/+	
2	5–7% +	< 5% −/+	
4	20%* +	25%* +	
**(B)**			
0.5	70%* +/++	<5% +	0
1	85%* ++	<5% +	
2	95%* ++/+++	85%* ++	
4	95%* ++/+++	25% +	

*For *p* ≤ 0.05

### The evaluation of HSP27

**Fig. 8:**
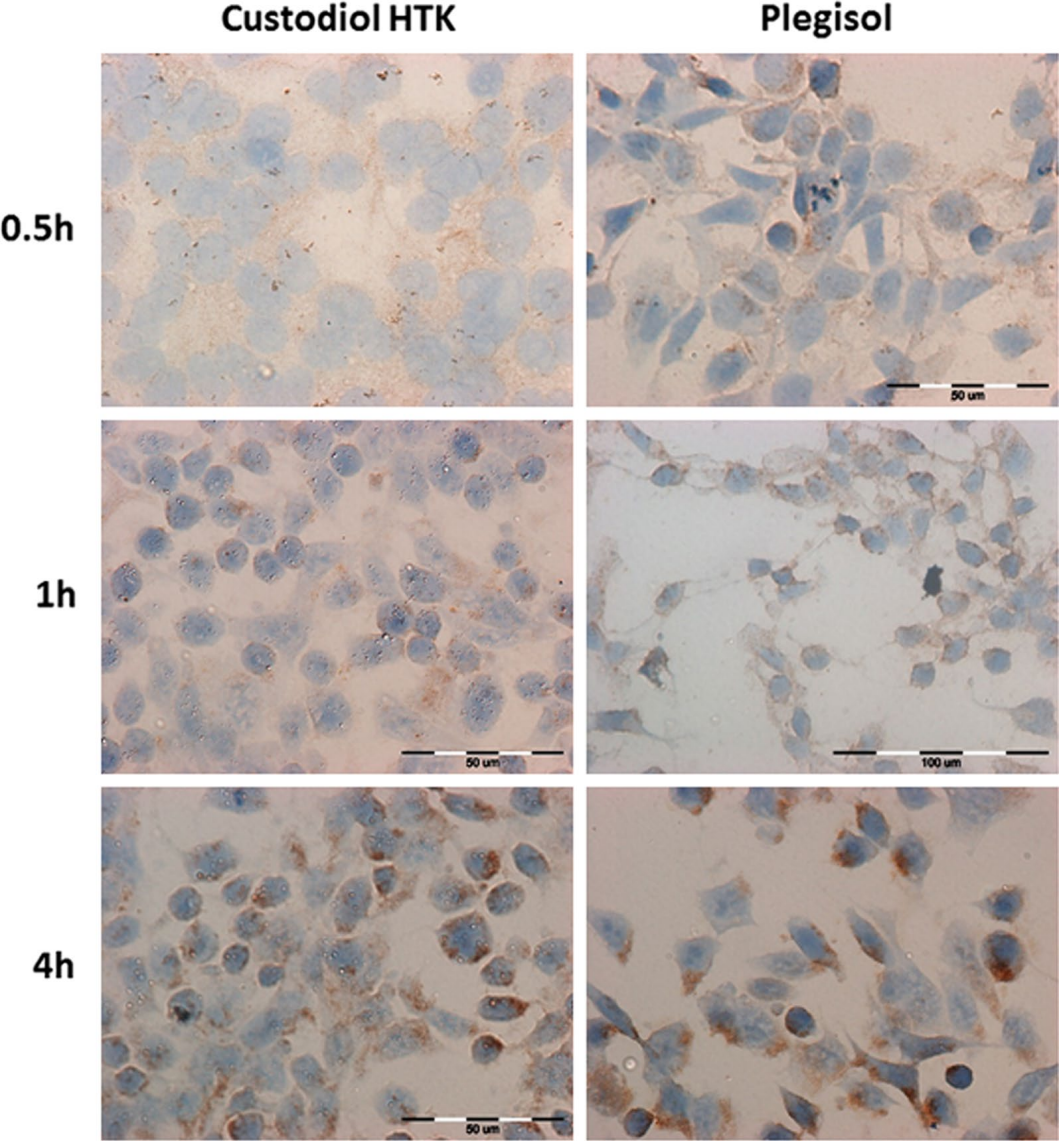
The evaluation of HSP27 immunocytochemical reaction in rat H9C2 cells after 0.5 h, 1 h and 4 h of incubation with Custodiol HTK or Plegisol

**Fig. 9:**
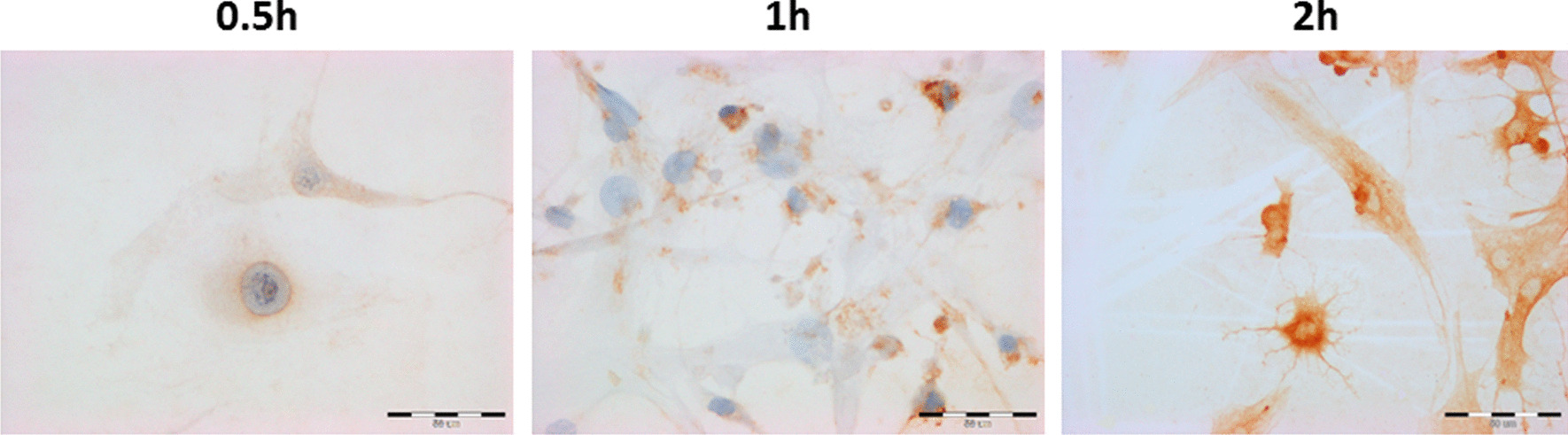
The evaluation of HSP27 immunocytochemical reaction in human HCM cells after 0.5 h, 1 h and 2 h of incubation with Plegisol

The results from the immunocytochemically stained reaction are presented in Fig. 8 for rat H9C2 after 0.5 h, 1 h and 4 h incubation with Custodiol HTK or Plegisol and in Table 4A. For HCM cells the obtained results are shown in Fig. 9 after 0.5 h, 1 h and 2 h of incubation with Plegisol and in Table 4B. Up to the first hour of incubation, the percentage of the stained cells was similar in both groups and significantly lower than control cells. After 2 h of incubation, Plegisol induced HSP27 expression more intensively than Custodiol HTK. After 4 h incubation with Plegisol, 70% of H9C2 were stained. Both cardioplegic solutions induced a decrease of HSP27 protein in HCM cells. Custodiol HTK has not activated the increasing expression of HSP27 in HCM cells. The HCM cells incubated with Plegisol demonstrated rising expression of HSP27 after 1 h and 2 h of incubation. After 4 h the reaction was not observed.

**Table 4:** The immunocytochemical evaluation of HSP27 expression in: (A) H9C2 cells, (B) HCM cells

Incubation time [h]	Custodiol HTK	Plegisol	Control cells
**(A)**			
0.5	< 5%* −/+	< 5%* +	45% ++
1	18%* +	20%* +	
2	20–12% +	40% +	
4	25%* +	70%* +/++	
**(B)**			
0.5	No reaction	<5% +	45% ++
1	No reaction	30% +/++	
2	No reaction	50%* +/++	
4	< 5%* +/++	no reaction	

*For *p* ≤ 0.05

## Discussion

The selection of an optimal strategy of heart protection is of utmost importance in modern cardiac surgery. According to the known comparative researches, there is no universal method of heart protection. The most popular one with depolarizing cardioplegic solutions allows to perform complicated surgical procedures, but it also has some significant limitations. Prolonging the ischemia period upsets cardiomyocytes’ natural mechanisms against ischemia/reperfusion injury even if the protection protocol fully complies. The cardiac cells affected by the pathological process are even more sensitive to the deleterious effect of ischemia, leading to unsatisfactory operative results of patients during acute myocardial infarct, with impaired systolic function [7] or pulmonary hypertension [8]. Although crystalloid cardioplegia was introduced in the 70’ of the last century is still an essential element in the whole cardiac operative protection strategy. There are a few well-known crystalloid solutions that use membrane depolarizing abilities of potassium ions. However, different ion concentrations and other added substances did not significantly influence the protective abilities of crystalloid cardioplegia, and the superiority of a specific solution has not been proven yet.

Numerous researchers compared blood and crystalloid cardioplegias [9–16], but only very few had compared different kinds of crystalloid solutions with unambiguous results. Kober et al. showed better protective abilities of Bretschneider solution in comparison to St. Thomas-2 [17]. On the other hand, T. Demmy et al. in the multicenter clinical trial indicated more structural protein release (cTn-I) and adverse events in the CABG-patients protected with Custodiol HTK [18]. Nevertheless, Cannata et al. did not find a correlation between the results of heart transplantation and the type of crystalloid cardioplegia [19].

According to the results of the clinical trials, it remains unclear if the quality of cardioprotection is associated more with the type of cardioplegia or other factors like protocol and cardioplegia delivery, patient morbidity and status of coronary vessels, a protocol of anesthesia, type of surgical procedure and finally the surgeon experience [1].

The present study considered cultured cells to be a helpful model allowing for standardized research protocol and objective cardioplegia assessment. Isolated cardiomyocytes are commonly applied as an explorative model in studies on ischemia and heart protection. Despite some disadvantages, Oldenburg et al. regarded cultured cardiomyocytes as a comfortable and economical example of an explorative research model. They indicated the possibility of performing reproducible experiments with a low dose of pharmacological agents under investigation. [20] The other group of Wei et al. applied H9C2 cells to examine the protective properties of NAC (N-acetylo-L-cystein) from ROS [21]. Dong et al. exposed H9C2 cells to chemical ischemia and demonstrated the protective effect of H_2_S by inhibition of protein kinase ERK1/2 [22]. Although cultured cardiomyocytes were not too often involved to compare cardioplegic solutions, Camilleri et al. were one of the first who confirmed the usefulness of cardiomyocytes to investigate crystalloid cardioplegia [23]. They proved lower toxicity of St. Thomas-2 as compared to classic Bretschneider solution on neonatal rat cardiomyoblasts which had been exposed to the chemical hypoxia.

Our study provoked ischemia by moving cells from the culture medium and plunged into cold cardioplegic solutions followed by incubation for 4 h simulating the intraoperative conditions. The presence of oxidative stress was proven by the increased level of oxidative stress markers. The chemical hypoxia was avoided to minimize its impact on the incubated cell.

The cytotoxicity index in MTT test on HCM did not decrease significantly within 4 h of incubation, confirming no influence of both investigated cardioplegia solutions on the activity of mitochondrial dehydrogenase of HCM. However, the cytotoxicity index dropped below 50% after 4 h of H9C2 incubation with Plegisol. The highest dehydrogenase level was induced after incubation of HCM with Custodiol HTK at 2 h of incubation, statistically different from Plegisol. The predominance of Plegisol, but not statistically different, was observed after 0.5 h of H9C2 incubation. On this basis, we may assume that Custodiol is less cytotoxic in comparison to Plegisol. Others have also applied cytotoxicity index in order to assess cytoprotection. Gomez et al. assessed the viability of isolated adult cardiomyocytes underwent stress factors analysis in the MTT testing, and revealed a direct cytoprotective effect of adenosine and hyperkalemic-cardioplegia [24]. Similarly, Drescher et al. used a cytotoxicity test to prove the cardioprotective property of hypothermia on H9C2 cells incubated in a mixture of cardioplegia and culture medium [25].

One of the best-known oxidative stress markers is MnSOD. In our research significant increase in MnSOD expression was observed, but not specific correlation between the intensity of reaction, the percentage of stained cells, and incubation time were found, neither no difference between Custodiol and Plegisol were noted. Previously, we examined the expression of MnSOD in the right atrium appendage of the cardioplegicaly arrested heart during coronary revascularization [26]. As we observed, the MnSOD expression increased in sections of the right atrium appendage by the rise of stain intensity and the percentage of cells with positive stain. Shlafer et al. showed better protective abilities of crystalloid cardioplegia after antioxidative enzyme supplementation (SOD, CAT) on isolated rabbit hearts [27]. Lee et al. proved the preventive role of Propofol in hypoxia/reoxygenation—induced apoptotic H9C2 cells [28]. On the other hand, essential differences were observed in the expression of iNOS in HCM. There was an observed higher intensity of the immunocytochemical reaction against iNOS in HCM cells than neonatal H9C2 cells. HCM are mature cells derived from the adult; thus, the mechanisms of antioxidative response are better developed. Custodiol HTK induced significantly stronger iNOS expression as compared to Plegisol. The iNOS expression is considered as an apoptotic marker. As a result, it can be assumed that Custodiol appears as less destructive to cell self-defense mechanism than Plegisol, preventing the reactive impact of ROS. The role of iNOS signaling in myocardial cells is very clear [29]. Inducible nitric oxide synthase activates after the external stimuli upon the presence of stress. The strong iNOS production may confirm myocardial protection [30]. Neonatal cells have these mechanisms weakly developed; thus, this type of cells may require more time to activate antioxidant defense.iNOS was successfully used in the past as myocardium ischemia markers by Jung et al. [31] and Heinzel et al. [32]. Also, Mayers et al. confirmed an increase in NOS activation during cardiac surgery with CPB [33].

The assessment of protein HSP27 also revealed better protective properties of Custodiol HTK. The decrease of HSP27 concentration has been instantaneous and maintained throughout the study in both cultures incubated with Custodiol HTK. The absence of HSP27 after 0.5 h of incubation proves better protective abilities of Custodiol. Giannesi el al. also noticed a correlation between HSP synthesis and necrotic myocardial markers (Troponin-I, Myoglobin, LDH, CK, CK-MB) activation after heart operations [34].

The level of lipid peroxidation was similar after incubation with both solutions. Our results corroborated sufficiently with Bical et al. and Zhang et al. examining various types of cardioplegia and different reperfusion techniques finding the similar intensity of peroxidation despite the protection method they used [35, 36].

Protein damage reflected defined as carbonyl groups concentration was very irregular. The increased level of protein concentration was observed in H9C2 cells at the longest time of exposition. On the contrary, in HCM cells carbonyl groups level has been nonspecific and not different from control cells, so it was challenging to select the solution of better protection from oxidation.

The in-vitro model of incubated cultured cardiomyocytes is not very popular in the field of cardiac protection. Superior iNOS and HSP27 expressions, and MTT test, proved better potential protective properties of Custodiol HTK. Nevertheless, in the RCT by Demmy et al. showed no clinical advantage. Moreover, Custodiol HTK in the one-dose protocol generated a higher cTn-I level 6 h after operation than multi-dosed Plegisol [18]. Thus, the in-vitro superiority of Custodiol HTK has not been confirmed by this clinical study. Similarly, a systematic review by Edelman et al. showed no clear superiority of Custodiol over other cardioplegic solutions in preoperative myocardial protection [37]. In the author’s opinion intra-operative myocardial protection is multifactorial when only one of those factors relates to a cardioplegia solution alone.

### Study limitations

The main limitation of our research is that in vitro studies results cannot be directly transferred into clinical practice without proper clinical assessment, optimally in a randomized fashion. However, they can deliver fundaments for myocardial protection improvement.

## Conclusions

Our in-vitro study reveals usefulness of cell cultures as a research model. Custodiol HTK—low-potassium cardiplegia appears to be less harmful and safer than Plegisol for maintaining cellular function.

## Data Availability

The datasets used and/or analyzed during the current study are available from the corresponding author on reasonable request.

## Abbreviations

CPB: Cardiopulmonary by-pass
H9C2: Rat cardiomyocytes
HCM: Human cardiomyocytes
MnSOD: Manganese superoxide dismutase
iNOS: Inducible nitric oxide synthase
HSP27: Heat shock protein 27
OS: Oxidative stress
ROS: Reactive oxygen species
MDA: Malondialdehyde
MTT: 3-(4,5-Dimethylthiazol-2-yl)-2,5-diphenyltetrazolium bromide (tetrazolium dye)
PBS: Phosphate-buffered saline
TCA: Trichloroacetic acid
TBA: Thiobarbituric acid
TBARS: Thiobarbituric acid reactive substances
DNPH: Dinitrophenylhydrazine
DAKO: Diaminobenzidine-H_2_O_2_
CABG: Coronary artery by-pass grafting
cTn-I: Cardiac troponin I
SOD: Superoxide dismutase
CAT: Catalase
